# Induction of neuro-protective/regenerative genes in stem cells infiltrating post-ischemic brain tissue

**DOI:** 10.1186/2040-7378-2-11

**Published:** 2010-05-28

**Authors:** Gokhan Yilmaz, J Steven Alexander, Cigdem Erkuran Yilmaz, D Neil Granger

**Affiliations:** 1 Department of Molecular and Cellular Physiology, Louisiana State University Health Science Center, Shreveport, LA, USA; 2 Department of Cell Biology and Anatomy, Sophie Davis School of Biomedical Education, CUNY, NY, USA

## Abstract

**Background-:**

Although the therapeutic potential of bone marrow-derived stromal stem cells (BMSC) has been demonstrated in different experimental models of ischemic stroke, it remains unclear how stem cells (SC) induce neuroprotection following stroke. In this study, we describe a novel method for isolating BMSC that infiltrate postischemic brain tissue and use this method to identify the genes that are persistently activated or depressed in BMSC that infiltrate brain tissue following ischemic stroke.

**Methods-:**

Ischemic strokes were induced in C57BL/6 mice by middle cerebral artery occlusion for 1 h, followed by reperfusion. BMSC were isolated from H-2 Kb-tsA58 (immortomouse™) mice, and were administered (i.v.) 24 h after reperfusion. At the peak of therapeutic improvement (14 days after the ischemic insult), infarcted brain tissue was isolated, and the BMSC were isolated by culturing at 33°C. Microarray analysis and RT-PCR were performed to compare differential gene expression between naïve and infiltrating BMSC populations.

**Results-:**

Z-scoring revealed dramatic differences in the expression of extracellular genes between naïve and infiltrating BMSC. Pair-wise analysis detected 80 extracellular factor genes that were up-regulated (≥ 2 fold, *P *< 0.05, Benjamini-Hochberg correction) between naïve and infiltrated BMSC. Although several anticipated neuroregenerative, nerve guidance and angiogenic factor (e.g., bFGF, bone morphogenetic protein, angiopoietins, neural growth factor) genes exhibited an increased expression, a remarkable induction of genes for nerve guidance survival (e.g., cytokine receptor-like factor 1, glypican 1, Dickkopf homolog 2, osteopontin) was also noted.

**Conclusions-:**

BMSC infiltrating the post-ischemic brain exhibit persistent epigenetic changes in gene expression for numerous extracellular genes, compared to their naïve counterparts. These genes are relevant to the neuroprotection, regeneration and angiogenesis previously described following stem cell therapy in animal models of ischemic stroke.

## Background

In various animal models of central nervous system (CNS) injury, bone marrow stromal cells (BMSC) have been reported to be effective in limiting tissue damage[1-4]. The therapeutic effects of stem cells have been attributed to their ability to release of a mixture of neurotrophins, growth factors, and other substances that induce restorative processes in post-ischemic brain tissue[1,5-8]. Although some studies have focused on the role of one or more potential neuroprotective factors that are released from BMSC, less effort has been made to evaluate the responses of BMSC once they infiltrate brain tissue following an ischemic insult[7,9].

Immortalized cell lines have long been used as a source of stem cells for in vivo studies of the therapeutic efficacy of stem cells in models of ischemic tissue injury[10-12]. While these cells have been shown to survive and differentiate in brain tissue and to afford some protection against ischemic stroke, they are difficult to recover from brain tissue to permit assessment of the phenotypic and genetic changes that underlie their protective actions. Immortalized BMSC isolated from the H-2Kb-tsA58 transgenic mouse express a gene for temperature sensitive conditional immortality that makes them a more suitable model for stem cell recovery in ischemic tissue[13]. Stem cells from this background exhibit stem cell marker characteristics for over a year[13] and can give rise to cells of the mesenchymal lineage [14]. A particularly advantageous characteristic of BMSC from the H-2 Kb-tsA58 transgenic strain, is that the cells are undifferentiated at 33°C, but will acquire a differentiated phenotype at 37°C to yield a large number of cells with stem cell properties.

Although it is widely accepted that stem cells administered in animal models of stroke selectively home to and infiltrate the site of brain injury[14,15], successful isolation and genetic evaluation of these cells after they have infiltrated the post-ischemic brain has not been reported to date. In this study, we employed immortalized BMSC from H-2 Kb-tsA58 mice to selectively isolate stem cells that infiltrate brain tissue and produce therapeutic benefit following focal ischemia and reperfusion. The infiltrating BMSC were probed using whole genome array and RT-PCR in order to identify genes that are persistently up- or down-regulated in the stem cells after their appearance in infarcted brain tissue.

## Methods

### Animals

All in vivo experiments were performed on male C57Bl/6J mice (WT; 6 to 8 weeks old) (Jackson Laboratories, Bar Harbor, Me). BMSC were isolated from either H-2Kb-tsA58 mice expressing temperature-sensitive SV40 large T antigen (Large T; CBA/ca × C57Bl/10 hybrid, Charles River Laboratories) or from WT mice. The experimental procedures employed in this study were approved by the Louisiana State University Health Sciences Center Institutional Animal Care and Use Committee and are in compliance with the guidelines of National Institutes of Health.

### BMSC isolation

Primary cultures of BMSC were obtained from WT or Large T mice as previously described[16]. Briefly, fresh complete bone marrow was harvested aseptically from the tibias and femurs and then cultured in Iscove's Modified Dulbecco's medium (IMDM) supplemented with 10% fetal bovine serum (FBS). BMSC isolated from Large T mice were cultured at 33°C for selective isolation of the immortalized cells. After 3 days of incubation, non-adherent cells were removed and cells tightly adhered to plastic were isolated and resuspended to fresh Iscove's Modified Dulbecco's medium in new flasks for further growth. By passage 3, less than 1% of cells were positive for CD11b and CD45 as assessed by flow cytometry, and the BMSC were stem cell antigen-1 (sca-1) positive (70%). Prior to use in the in vivo model, BMSC were harvested using a non-enzymatic dissociation solution (Sigma Chemicals, St Louis), centrifuged at 1000 × g, filtered through a 70 um cell strainer (BD, Falcon), and resuspended in PBS (pH 7.4). 2 × 10^6 ^viable WT or Large T BMSC(in 150 μl of PBS) or PBS (150 μl) were administered intravenously at 24 hours after the induction of cerebral ischemia.

### Middle cerebral artery (MCA) occlusion and reperfusion (MCAo/R)

The mice were anesthetized by intraperitoneal injection of ketamine (50 mg/kg) and xylazine (2.5 mg/kg). Transient (60 minutes) focal cerebral ischemia was induced by occlusion of the left middle cerebral artery (MCAo) using a modification of intraluminal filament method. Briefly, the blunted tip of a 6-0 nylon monofilament was advanced to the level of the carotid bifurcation via the internal carotid artery until light resistance was felt. The distance from the nylon thread tip to the internal carotid artery-pterygopalatine artery bifurcation was slightly greater than 6 mm, and the distance to the bifurcation of the internal and external carotid arteries was slightly less than 9 mm. The monofilament was removed after 60 minutes of occlusion. In the sham group, these arteries were visualized but not disturbed. Ischemia and reperfusion (I/R) were verified using a Laser Doppler flowmeter probe (MSP300XP, AD Instruments Inc.) attached to the left parietal cranium. At the end of experiments, mice were killed with a lethal dose of pentobarbital (150 mg/kg, i.p.). The brains were immediately removed, and then stained with 2% 2, 3, 5-triphenyltetrazolium chloride to confirm the production of an infarct.

### Neurological score

In another set of experiments, the therapeutic effects of SC were assessed in mice receiving either 2 × 10^6 ^viable WT SC or Large T SC (in 150 μl of PBS) or PBS (150 μl) intravenously at 24 hours after the induction of cerebral ischemia. The neurological outcome was assessed at 1, 7 and 14 days after administration of SC using a 5-point scale neurological deficit score (0 = no deficit, 1 = failure to extend right paw, 2 = circling to the right, 3 = falling to the right, 4 = unable to walk spontaneously)[17].

### Recovery of Large T BMSC from ischemic brains

Mice were sacrificed 14 days after BMSC administration with a lethal dose of pentobarbital (150 mg/kg, i.p.). Ischemic hemispheres were removed from recipient mice that received either WT or Large T BMSC. The infarcted cerebral hemisphere was cut into small pieces and incubated with 2% collagenase at 37°C for 2 hours. The collagenase treated hemispheres were centrifuged at 1500 RPM for 10 min. The supernatants were discarded and tissues were resuspended in IMDM with 10% FBS and 1% streptomycin/penicillin. The suspensions were filtered through a 70-μm cell strainer (BD Falcon) and the filtered fractions were plated into 75 cm^2 ^flasks, cultured at 33°C in a mixture of 5% carbon dioxide and 95% oxygen, with the media replaced as needed. The cells isolated from ischemic brain tissue of mice receiving Large T BMSC reached confluency in 7 - 10 days. However, no cell growth was detected when infarcted tissue derived from mice receiving either WT BMSC or saline was cultured at 33°C under identical culture conditions.

### Immunostaining of Large T antigen (TAg)

Immortalized stem cells (~10^4 ^cells) were adhered onto 1.2 cm diameter coverslips by centrifugation at 1,500 × g for 15 min in culture medium. Coverslips were then fixed in 1% paraformaldehyde in PBS (30 min), and extracted in 0.2% Triton-X100/PBS (5 min). Permeabilized coverslips were incubated for 1 h in 75 ul of mouse anti-SV40 large TAg (1:150), (pab416; ts A58, AbCAM, Cambridge, MA) in 0.1% milk powder in PBS (MPBS) at 25°C. Coverslips were washed 3-times in 0.1% MPBS, and then incubated with 75 ul of goat anti-mouse fluorescein conjugated antibody (1:50) in MPBS (1 h). After 3 washes in 0.1% MPBS, coverslips were mounted in 1:5 diluted Vectashield/DAPI (Vector Labs, Burlingame, CA) and sealed with nail polish. Using fluorescence microscopy (Olympus AX70 microscope), the BMSC were examined for fluorescein (SV40 large Tag) and nuclei (DAPI), and the images captured with a Nikon Coolpix camera.

### Gene microarray

Pair-wise gene expression analysis was performed to compare the differences in gene expression patterns of naïve Large T (cell population injected into mice) with the Large T cell population isolated from infarcted tissue (iLarge T). RNA was extracted from cells using QIAshredder (Qiagen, Hilden, GmbH) and an RNeasy mini kit (Qiagen, Maryland), according to manufacturer's directions. RNA integrity was assessed by electrophoresis on the Agilent 2100 Bioanalyzer (Agilent Technologies, Palo Alto, CA). Double-stranded cDNA was synthesized from approximately 7 ug total RNA, using a Superscript cDNA Synthesis Kit (Invitrogen, Carlsbad, CA) in combination with a T7-(dT)_24 _primer (Proligo, Boulder, CO). Biotinylated cRNA was transcribed in vitro using the BioArray High Yield RNA Transcript Labeling Kit (ENZO Biochem, New York, NY) and purified using the GeneChip Sample Cleanup Module (Affymetrix, Santa Clara, CA). Twenty micrograms of purified cRNA was fragmented by incubation in fragmentation buffer (200 mM Tris-acetate, pH 8.1, 500 mM potassium acetate, 150 mM magnesium acetate) at 94°C for 35 minutes and chilled on ice. Ten micrograms of fragmented biotin-labeled cRNA was hybridized to the Mouse Genome 430 2.0 Array (Affymetrix), interrogating over 39,000 transcripts. Arrays were incubated for 16 hr at 45°C with constant rotation (60 rpm). The arrays were washed and then stained for 10 min at 25°C with 10 ug/mL streptavidin-R phycoerythrin (Vector Laboratories, Burlingame, CA) followed by 3 ug/mL biotinylated goat anti-streptavidin antibody (Vector Laboratories) for 10 minutes at 25°C. Arrays were then stained once again with streptavidin-R phycoerythrin for 10 min at 25°C. After washing and staining, the arrays were scanned using a GeneChip Scanner 3000. Pixel intensities were measured, expression signals were analyzed and features extracted using the commercial software package GeneChip Operating Software 1.2 (*Affymetrix*).

Three independent sets of experiments were performed, each containing RNA samples pooled from BMSC populations isolated from 3 ischemic hemispheres or 3 sets of naïve Large T SC. Data mining and statistical analysis were performed with genesifter.net software. A two-fold or more change in gene expression with an unpaired t-test corrected with the Benjamini-Hochberg procedure[18], and a p < 0.05 was considered significant and used for further analysis. The Z-score was used to detect the most affected gene ontology families, with a high Z-score indicating a highly affected pathway[19]. Genes related to molecular function, localization and biological processes were analyzed by gene ontology detecting software http://www.genesifter.net.

### Real time quantitative PCR

Total RNA extracted from naïve and iLarge T SC was used as a template for cDNA synthesis. For each sample, 100 ng of total RNA was used as a template for cDNA synthesis. The reverse transcription reaction was performed in master mix containing RT buffer, MgCl_2_, dNTPs, random hexamers, RNase inhibitor and multiscribe reverse transcriptase (Applied Biosystems, Foster City, CA). Incubations were performed in a Mastercycler-personal (Eppendorf, Westbury, NY) for 10 min at room temperature followed by 60 min at 42 C and 5 min at 95 C. For quantitative real-time PCR analysis the primers listed in Table S1, Additional file 1 were used (Realtime Primers, Elkins Park, PA). Fast SYBR Green master mix was used for amplification and detection. Reactions were performed in triplicate using an ABI Prism 7900 Sequence Detection System. Raw data were analyzed using the ABI Prism Sequence Detection 1.9.1 software. The comparative Cr method for relative quantification of gene expression was used to determine expression levels for target genes. Beta-actin was used as a housekeeping gene.

## Results

### Large-T BMSC improve neurological outcome after MCAO

**Figure 1:**
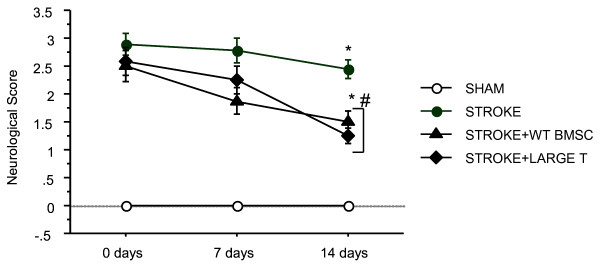
Neurological scores of sham, stroke, stroke +WT BMSC and stroke + Large T BMSC groups over a two week period after stroke or sham surgery. * represents significant difference from sham group (*p *< 0.05), # represents significant difference from stroke group (*P *< 0.05), one-way ANOVA with Tukey's post-hoc test.

Intravenous administration of 2 × 10 ^6 ^Large T BMSC after 1 hour of cerebral ischemia and 24 hrs reperfusion blunted the neurological deficits (improved the neurological scores) normally noted in untreated mice two weeks thereafter (Figure 1). The protective effect of Large T BMSC was comparable to that observed in mice treated with BMSC isolated from wild type mice. Even though an improvement was observed with administration of WT BMSC or Large T BMSC at 7 days after ischemic stroke, a statistical difference was observed only at 14 days after BMSC administration. No difference in infarct volumes was observed among untreated stroke group versus the groups that received either WT-BMSC or Large-T BMSC (data not shown). These results indicate that Large T BMSC exhibit similar neuroprotective properties as BMSC isolated from WT mice.

### Viable Large-T BMSC can be recovered from post-ischemic brain tissue

**Figure 2:**
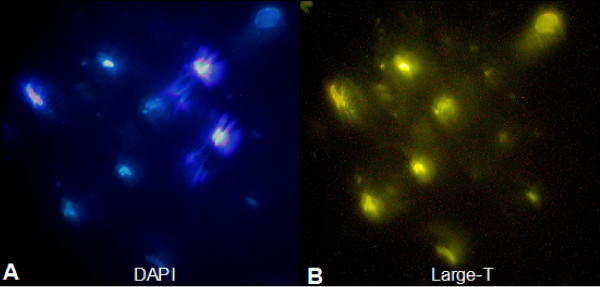
**Large T BMSC isolated from ischemic brain**. A. Nuclear DAPI Staining, B. Large T immunostaining of the same sample.

In order to analyze potential phenotypic changes in BMSC that are recruited into post-ischemic brain, we devised a novel approach for tissue isolation of BMSC that capitalized on the ability of Large T BMSC to grow under culture conditions at 33°C. Using this approach, we found that Large T BMSC harvested from post-ischemic brain tissue and then cultured at 33°C exhibit cell growth and colony formation, while WT BMSC did not exhibit these responses under identical experimental conditions. Immunocytochemical staining confirmed the presence of intracellular Large T antigen in all cells isolated using this procedure (Figure 2)

### BMSC isolated from post-ischemic brain tissue exhibit an altered gene expression pattern compared to their naive counterparts

**Figure 3:**
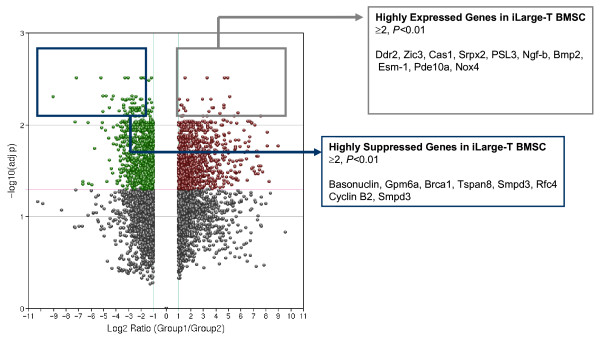
**Volcano graph of gene expression changes in BMSC isolated from ischemic brain (Group1) versus naïve BMSC (Group2)**.

**Figure 4:**
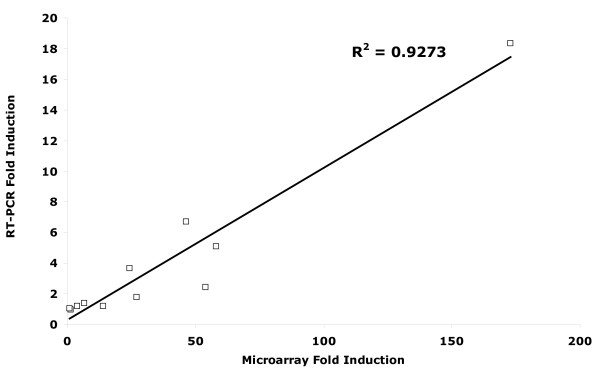
**Fold-change in gene expression detected by RT-PCR versus microarray**.

The GeneChip^® ^Mouse Genome 430 2.0 array, covering over 39,000 transcripts, was used to compare gene expression between BMSC isolated from infarcted tissue (iLarge T BMSC) and naïve Large T SC. A pair-wise analysis of three independent experiments revealed a dramatically altered gene expression profile in iLarge T BMSC. Using filtering criteria of a two-fold or more change in gene expression with an unpaired t-test corrected with Benjamini-Hochberg procedure[18] (P < 0.05) a list of 1885 differentially expressed genes were detected (Figure 3). Of this total, 995 genes from iLarge T BMSC exhibited reduced expression (Table S2, Additional file 2), while 890 genes showed increased expression (Table S3, Additional file 3). Among the highly up-regulated genes (adjusted p < 0.01), endothelial specific cell molecule-1, bone morphogenic protein-2, nerve growth factor beta, olfactomedin-1 were detected. The gene microarray was validated with RT-PCR. A strong correlation (r^2 ^= 0.93) between mRNA detected by RT-PCR and the gene microarray results was observed (Figure 4). The data discussed in this publication have been deposited in NCBI's Gene Expression Omnibus[20] and are accessible through GEO Series accession number GSE21393 http://www.ncbi.nlm.nih.gov/geo/query/acc.cgi?acc=GSE21393.

### Genes in Large-T BMSC that encode factors released into the extracellular space are highly induced by ischemia-reperfusion

**Figure 5:**
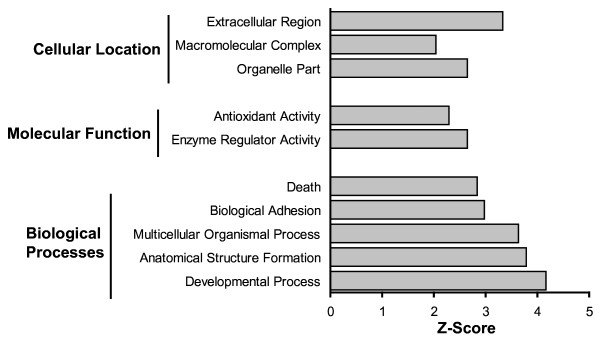
**Z-scores of gene ontology groups that changed in BMSC isolated from ischemic brain**.

A gene ontology analysis was performed on Large T BMSC using Genesifter.net ontology tools. Z-scoring was used to identify the most affected pathways in isolated BMSC. A positive Z-score indicates that more genes than expected fulfilled the criterion for altered expression in a certain group or pathway; therefore, that group or pathway is likely to be affected by the imposed condition (e.g., ischemia)[21]. Z-scores were detected for up-regulated transcripts related to biological processes, cellular location and molecular function (Figure 5). A very high Z-score was detected for genes targeting the extracellular region.

**Figure 6:**
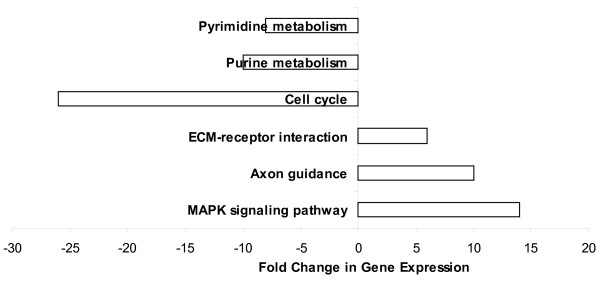
**Changes in KEGG pathways in BMSC isolated from ischemic brain**. Changes filtered for parameters Z-score > 2 and 10 or more changed genes in related group.

**Figure 7:**
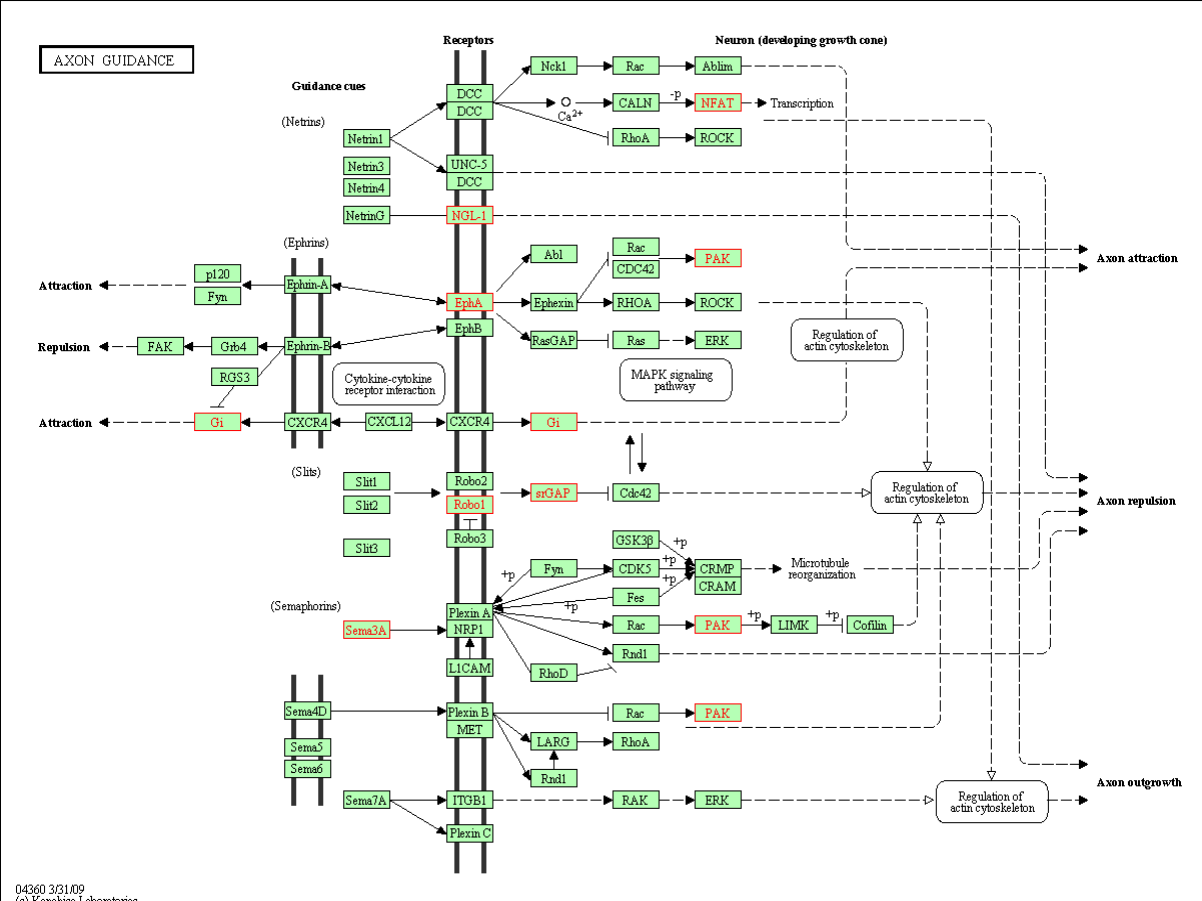
**Axon guidance KEGG pathway affected in BMSC isolated from ischemic brain**. Genes affected represented in red.

Since previous reports suggest that stem cells likely exert their beneficial effects through the secretion of trophic factors that enhance brain repair[22]^, ^[23], we focused our analysis on the extracellular factors that exhibit altered expression in BMSC isolated from post-ischemic brains. Eighty extracellular factors were increased in iLarge T BMSC, compared to their naïve counterparts (Table S4, Additional file 4). Among the extracellular factors affected, some have previously been reported as secreted by BMSC (red highlighting in Table S4, Additional file 4), while others (blue highlighting in Table S4, Additional file 4) have been associated with brain ischemia. A KEGG (Kyoto Encyclopedia of Genes and Genomes) pathway analysis was performed by setting the absolute Z-score above 2 and number of genes in a set as ≥ 10 (Figure 6). This analysis revealed an up-regulation in genes related to the mitogen-activated protein kinase (MAPK) and axon guidance pathways, and a down-regulation of genes in cell division-related pathways. The KEGG diagram for axon guidance pathways in iLarge T BMSC were consistent with activation of a number of relevant genes, including Eph receptors (mediate neuronal branching), GTPase activators, and semaphorins (Figure 7).

## Discussion

Stem cell therapy has received much attention as a potential post-injury intervention to repair stroke-damaged brain tissue. While there is limited clinical evidence that convincingly demonstrates stem cell mediated symptomatic relief in individuals who have suffered a stroke[24], animals studies suggest that stem cell therapy has the potential to reverse some of the behavioral deficits that result from ischemic stroke[1,3,4,25,26]. The success in animal models has led several laboratories to focus on potential mechanisms that may underlie the neuroprotective effects of stem cell therapy. A limitation of these mechanistic studies is an inability to isolate and characterize the population of stem cells that infiltrate post-ischemic brain tissue. Single cell isolation methods, such as laser capture, have provided some insights, but the low yield and poor survival of the recovered stem cells limits the utility of this approach. Here, we introduce a novel approach for selective isolation of BMSC from infarcted brain tissue. This approach yields viable infiltrating stem cells of sufficient number to allow for a detailed analysis of changes in gene expression.

Stem cells that infiltrate a brain infarct are believed to synthesize and secrete growth and guidance factors that are protective and orchestrate tissue recovery following stroke. However, the epigenetic changes that occur in these infiltrating stem cells due to environmental cues produced by the ischemic insult remain poorly understood. The identification of genes that exhibit significantly altered expression by protective tissue-infiltrating stem cells represents a powerful approach for discovery of factors produced by stem cells that enable them to mediate tissue restitution and repair following an ischemic stroke. Our analysis revealed that the expression of a large number of genes is dramatically and persistently increased in BMSC harvested from the ischemic infarct, when compared to their 'naïve' (non-migrated) precursors. Interestingly, many of the genes whose messages were most dramatically increased (> 100 fold) appear to play important roles in neural patterning, remodeling, adhesion and angiogenesis. Many other genes not previously associated with neuroprotection were also dramatically increased and these genes may play important supporting roles in tissue recovery from stroke.

Different mechanisms have been proposed to explain the improved neurological outcome following stem cell treatment, including trans-differentiation into neural lineages, cell fusion, and neuroprotection through trophic support. A role for neuroprotection mediated by released trophic factors is supported by reports that describe the wide array of neuroprotective factors released from stem cells that could lead to improved neurological outcome after stroke by promoting physiological responses such as angiogenesis and/or neurogenesis, and inhibition of scar formation. More direct support is provided by Qu and coworkers[7], who identified, using gene microarray, the factors secreted by BMSC after exposure to ischemic brain extract. Their comparison of the gene expression profiles between naive BMSC and ischemic extract-exposed BMSC revealed an intense up-regulation of genes that encode extracellular factors, such as fibroblast growth factor-2, epidermal growth factor, nerve growth factor-beta, insulin-like growth factor 1, VEGF-A, transforming growth factor, beta 1, and brain derived neurotrophic factor[7]. Our analysis of BMSC that infiltrate post-ischemic brain tissue demonstrated gene activation for most of the same trophic agents (Table S4, Additional file 4). In our study, the expression of VEGF-A, VEGF-C and TGF-beta were all increased in BMSC isolated from post-ischemic brains, but these responses did not reach statistical significance in the microarray assay. However, consistent with previous reports[27], we found that angiopoietin-1 and 4 were highly expressed in BMSC isolated from post-ischemic brains. Hence, our results provide strong support for the contention that within the post-ischemic environment, BMSC release angiogenic and neurotrophic factors that may mediate the neuroprotection observed following stem cell therapy.

Ddr2 (CD167b), a collagen adhesive receptor that participates in matrix integrin signaling[28], was increased 145-fold over naïve BMSC in our study, suggesting that Ddr2 matrix signals may contribute to post-stroke remodeling. ZIC-3 (Zinc finger protein-3) message was increased 26-fold over naïve BMSC. ZIC-3, a member of the C2H2-type zinc finger protein family, is a nuclear transcription factor that functions in left-right body axis alignment, and is a 'pluripotency' factor expressed during cell regeneration[29]. Zic3 also interacts with BMP and FGF signaling to direct neural cell programming[30]. We also found that infiltrating BMSC expressed 195-fold more cytokine receptor-like factor-1 (Crlf-1, cytokine-like factor 1, CLF-1, CRLM-3, cytokine receptor like molecule 3, NR6) transcript than naïve BMSC. CRLF1 forms a heterodimeric complex with cardiotrophin-like cytokine factor 1 (CLC-1), and the Crlf-1/CLC-1 heterodimer competes with ciliary neurotrophic factor for binding to the ciliary neurotrophic factor receptor (CNTFR) complex. CrLF-1 is a cytokine ligand related to IL-12 that supports differentiation and survival of a wide range of neural cell types during embryonic development and in adult neural tissues[31]. CrLF-1 mRNA is up-regulated by inflammatory cytokines e.g. TNF-α, IL-6, and IFN-γ which are elevated in post-ischemic brain tissue[32].

Expression of FAM19A5 (also 'TAFA5') transcript was elevated 164-fold in brain penetrating BMSC, compared to naïve BMSC. FAM195 is a novel neuropeptide that is highly expressed in the CNS, particularly in hypothalamic paraventricular nuclear vasopressin and oxytocin cells[33]. FAM19a5 has been proposed to regulate brain fluid balance, and elevated levels of TAFA5 may thus help to control cerebral edema after stroke. Brain penetrating stem cells were found to express 101-fold more transcript for osteopontin (OPN) compared to naïve BMSC. OPN and thrombin generated OPN-peptides have all been shown to confer protection in stroke models[34,35]. Bayless has reported that OPN interacts with αvβ3 integrin and binds endothelial and smooth muscle cells in an RGD motif dependent manner[36]. OPN binding to α4β1 integrin has been implicated in the organization of endothelial cells in the developing vasculature, the extravasation of immune cells into tissues, and the emigration of neuroblasts[37]. Fibroblast growth factor-7 (FGF7 or KGF), which was increased 173-fold in our study, is known to mediate cell proliferation and motility, protect against cell death,[38] and has been shown to limit ischemia-induced neuronal death[39]. We also found a 44-fold increase in olfactomedin-1 (Olfm1), which has been implicated in neuronal differentiation, axon extension and cell survival[40].

Several genes that are linked to the Wnt signaling pathway were also profoundly upregulated in infiltrating BMSC. Glypican-1 (a GPI-anchored heparin sulfate proteoglycan) which interact with and suppresses hedgehog, stimulates the Wnt pathway, and binds BMP and FGF[41], exhibited a 155-fold increase. Dickkopf-2 (DKK2) expression was elevated 145-fold. Its homologue, Dickkopf-1 is a Wnt antagonist that contributes to neuronal apoptosis following brain ischemia[42]; DKK1 has been described as a target for treatment in neurodegenerative disorders e.g. beta-amyloid deposition, epilepsy, excitotoxicity. While DKK-1, (and Dkk4) block Wnt signaling, DKK2 and DKK3 do not,[43] and in some systems DKK2 actually synergizes with Wnt signals[44,45]. Therefore, in the setting of stroke recovery, elevated levels of secreted DKK2 might limit neuronal apoptosis to preserve neuron survival and improve tissue integrity.

Sushi-repeat containing protein (SRPX2), a ligand for urokinase-type plasminogen activator that interacts with cathepsins B and ADAMTS4 to control extracellular matrix remodeling[46], was elevated 33-fold in infiltrating BMSC compared naïve BMSC. SRPX2 participates in cell migration and adhesion through regulation of FAK phosphorylation[47]. It also contributes to the modulation of endothelial remodeling in angiogenesis[48], which may be a factor in the enhanced cerebral angiogenesis that is associated with stem cell therapy in ischemic brain disorders.

Several gene messages were found to be highly suppressed in BMSC isolated from the ischemic brain. Among these, basonuclin (Bnc1), a zinc-finger protein that is highly expressed in early keratinocytes[49], was down regulated 523-times. Down-regulation of this message may indicate a change in cell lineage fate within the ischemic environment. Glycoprotein m6a (Gpm6a) expression (reduced 157-fold) is associated with neuronal development and migration, and high levels of Gpm6a in stem cells has been linked to enhanced neuronal cell differentiation and migration[50]. We also noted a 34-fold suppression of breast cancer 1 gene (Brca1), a nuclear phosphoprotein that plays a role in maintaining genomic stability and in tumor suppression. Brca1 has been implicated in preventing apoptosis in early neuronal progenitors[51] and its expression in adult life is associated with Alzheimer's disease[52]. Smpd3 (reduced 24-fold) catalyzes the hydrolysis of sphingomyelin to form ceramide and phosphocholine. Ceramide mediates apoptosis and regulates the cell cycle by acting as a growth suppressor in confluent cells. Smpd3 also mediates cellular responses to IL-1ß and TNFα [53]. Tetraspanin 8 (Tspan8 or Tm4sf3) (reduced 23-fold), a member of the transmembrane 4 superfamily, is known to mediate signal transduction events that contribute to the regulation of cell development, activation, growth and motility[54]. Replication factor C (Rfc4) (9-fold reduction), which is required in the elongation of primed DNA templates by DNA polymerase δ and DNA polymerase ε[55], is believe to ensure error-free proliferation of stem cells at early phases of cell growth.

Astrocytes form glial scars along ischemic lesions and produce proteoglycans that inhibit axonal growth[56]. Suppression of inhibitory factors by cell-based therapies leads to axonal growth that correlate with improved functional outcome after stroke[57]. For example, it has been reported that bone-marrow mesenchymal cells reduce the expression of axonal-growth inhibitory proteins that are released by astrocytes, thereby allowing axon formation in the ischemic brain[58]. In our study, BMSC harvested from post-ischemic brain tissue exhibited a persistent and altered expression of several of genes that have been implicated in the regeneration and guidance of axons (Figure 7). Among these genes, Robo-1, EphA4, Pak1 and SLIT-Robo RhoGTPase activating protein exhibited the most significant up-regulation, while the expression of semaphorin 3D and ephrin-B1 were significantly reduced. These findings support the role of BMSC re-programming in axon formation and guidance following ischemic stroke.

Several studies suggest that stem cells can also attenuate immune responses of the host[59-61]. Supporting this view are changes in gene expression related to immune processes in human BMSC transplanted into the mouse hippocampus 1 day after global ischemia[9]. The transplanted BMSC exhibited a change in gene ontology groups for carbohydrate binding, cell adhesion, basement membrane as well as antigen presentation and processing. The results of our analysis suggest that the changes in immune-related gene processes were small in the BMSC that infiltrate ischemic brain tissue, particularly in comparison with the responses noted for genes related to axon guidance, MAPK pathway and the cell cycle. While an explanation for the different gene expression responses between the two studies is not readily apparent, it may result from differences in ischemic model, route of administration of BMSC, and/or brain region studied.

## Conclusions

In conclusion, using a novel approach for BMSC isolation from postischemic brain tissue, we found that BMSC assume a new and very different genetic profile that favors the secretion of numerous extracellular factors into ischemic brain tissue that have the potential to facilitate neuroprotective responses such as angiogenesis, and axonal guidance and regeneration. Our findings may help to explain the neuroprotective effects previously proposed for stem cells in ischemic stroke.

## Competing interests

All authors declare no conflict of interest. This work was supported by a grant from the National Heart Lung and Blood Institute (R01 HL26441-29).

## Authors' contributions

GY designed the study, performed animal and cell culture experiments, analyzed data, and wrote the paper. JSA generated H-2Kb-tsA58 stem cells, analyzed gene array data and wrote the paper. CEY performed immunohistochemistry and flow cytometry. DNG designed the study, provided lab facilities, helped to interpret data and wrote the manuscript. All authors read and approved the final manuscript.

## Supplementary Material

Additional file 1 **Table S1**. RT PCR primers Click here for file

Additional file 2 **Table S2**. Down-regulated transcripts in isolated BMSC from ischemic brain. Click here for file

Additional file 3 **Table S3**. Up-regulated transcripts in isolated BMSC from ischemic brain. Click here for file

Additional file 4 **Table S4**. Extracellular factors that are expressed in BMSC isolated from ischemic regions compared to naïve BMSC. Click here for file
