# Supplementary material for: Induction of neuro-protective/regenerative genes in stem cells infiltrating post-ischemic brain tissue
Source: Exp Transl Stroke Med. 2010 May 28;2:11. doi: 10.1186/2040-7378-2-11 (PMC2893124; doi:10.1186/2040-7378-2-11)
Supplement: Additional file 1 — Table S1. RT PCR primers [file 2040-7378-2-11-S1.DOC]

**Supplementary Table 1.**

**Real Time PCR Primers**

| **Gene** | **Accession no.** | **Forward** | **Reverse** |
| --- | --- | --- | --- |
| Angiopoietin 4 | NM_009641 | GACACAATCCAGAACCAGAC | CAGTGAGTTCTCCAGCATTT |
| Nerve growth factor beta | NM_013609 | AAGCTCACCTCAGTGTCTGG | TTTAGTCCAGTGGGCTTCAG |
| Bone morphogenic protein 2 | NM_007553 | TGAACACAGCTGGTCACAGA | ACTCCCCATGGCAGTAAAAG |
| Glial cell line derived neurotrophic factor | NM_010275 | CTCTAGCTCTTGGGGGAATC | ACGACCGAGACATCAGAGAG |
| Brain derived neurotrophic factor | NM_007540 | GGTGCAGAAAAGCAACAAGT | GCACAAAAAGTTCCCAGAGA |
| Insulin-like growth factor 2 | NM_010514 | TCACAGGTCAACTTGCAGAA | GCTCCCGTTATGTAGGGAAT |
| Fibroblast Growth Factor-7 | NM_008008 | AAAGGGGATTCCTGTCAAAG | GCAAGTCCAGCTTGATCAGT |
| Endothelial cell-specific molecule 1 | NM_023612 | TTCTCAAACCTTGCCAACAG | GACAAAACCTAACAGCCGGT |
| Tissue inhibitor of Metalloproteinease-2 | NM_011594 | CGCAGATATCCGGTACGCCTA | CACAAGCCTGGATTCCGTGG |
| S100B | NM_009115 | CAGAGACCCTTAATTCCCCA | GTTCCTGGAGGACAGAGGAG |
