# Supplementary material for: Induction of neuro-protective/regenerative genes in stem cells infiltrating post-ischemic brain tissue
Source: Exp Transl Stroke Med. 2010 May 28;2:11. doi: 10.1186/2040-7378-2-11 (PMC2893124; doi:10.1186/2040-7378-2-11)
Supplement: Additional file 4 — Table S4. Extracellular factors that are expressed in BMSC isolated from ischemic regions compared to naïve BMSC. [file 2040-7378-2-11-S4.DOC]

**Table 4** Extracellular factors that are expressed in BMSC isolated from ischemic regions compared to naïve BMSC.

| **Gene Name** | **Gene ID** | **Ratio** | **adj. *P*-value** |
| --- | --- | --- | --- |
| Cytokine receptor-like factor 1 (Crlf1), mRNA | Crlf1 | 194.98 | 0.007999 |
| **Fibroblast growth factor 7 (Fgf7), mRNA [39]** | **Fgf7** | **173.42** | **0.028064** |
| Family with sequence similarity 19, member A5 (Fam19a5), mRNA | Fam19a5 | 164.47 | 0.022009 |
| Glypican 1 (Gpc1), mRNA –axon guidance- | Gpc1 | 155.34 | 0.022009 |
| Dickkopf homolog 2 (Xenopus laevis) (Dkk2), mRNA | Dkk2 | 145.54 | 0.033978 |
| Endothelial cell-specific molecule 1, mRNA (cDNA clone MGC:28355 IMAGE:4018658) | Esm1 | 138.69 | 0.010088 |
| Transcribed locus | - | 126.32 | 0.029095 |
| **Osteopontin (OPN)[37]** | **Spp1** | **101.64** | **0.015096** |
| **Tissue factor pathway inhibitor 2, mRNA (cDNA clone MGC:13885 IMAGE:4023742)[9, 62]** | **Tfpi2** | **80.13** | **0.015493** |
| Masp3 mRNA for MBL-associated serine protease-3 | Masp1 | 60.33 | 0.024376 |
| **Glial cell line derived neurotrophic factor (Gdnf), mRNA [7]** | **Gdnf** | **58.33** | **0.032447** |
| RIKEN cDNA 1500015O10 gene (1500015O10Rik), mRNA | 1500015O10Rik | 57.42 | 0.014596 |
| **Bone morphogenetic protein 2 (Bmp2), mRNA[7]** | **Bmp2** | **54.9** | **0.007380** |
| Olfactomedin 1, mRNA (cDNA clone MGC:35933 IMAGE:5036675) | Olfm1 | 44.66 | 0.007999 |
| Sushi-repeat-containing protein, X-linked 2 (Srpx2), transcript variant 1, mRNA | Srpx2 | 33.72 | 0.004922 |
| Insulin-like growth factor binding protein 6, mRNA (cDNA clone MGC:14073 IMAGE:4224052) | Igfbp6 | 30.62 | 0.019522 |
| C1q-like 3 (C1ql3), mRNA | C1ql3 | 26.22 | 0.017747 |
| Angiopoietin-related protein-2 mRNA,complete cds | Angptl2 | 26.09 | 0.014596 |
| Ribonuclease, RNase A family 4, mRNA (cDNA clone MGC:11599 IMAGE:3967265) | Rnase4 | 25.17 | 0.012528 |
| Integrin, beta-like 1 (Itgbl1), mRNA | Itgbl1 | 24.78 | 0.024358 |
|  |  |  |  |
| **Gene Name** | **Gene ID** | **Ratio** | **adj. *P*-value** |
| **Nerve growth factor, beta, mRNA (cDNA clone IMAGE:4190781)[7]** | **Ngf** | **24.39** | **0.006472** |
| Angiopoietin-like 4, mRNA (cDNA clone MGC:35885 IMAGE:5137159) | Angptl4 | 21.86 | 0.010088 |
| Collagen, type X, alpha 1 (Col10a1), mRNA | Col10a1 | 19.43 | 0.038394 |
| Latent transforming growth factor beta binding protein 2 (Ltbp2), mRNA | Ltbp2 | 19.16 | 0.033421 |
| NP mRNA for neuropsin | Klk8 | 18.93 | 0.013183 |
| **Angiopoietin 1 (Angpt1), mRNA[27]** | **Angpt1** | **17.12** | **0.026503** |
| Tenascin C (Tnc), mRNA | Tnc | 14.82 | 0.020695 |
| **S100 protein, beta polypeptide, neural (S100b), mRNA[63]** | **S100b** | **14.03** | **0.044087** |
| **Thrombospondin 2, mRNA (cDNA clone IMAGE:3583417)[64]** | **Thbs2** | **13.67** | **0.043275** |
| C1q and tumor necrosis factor related protein 3, mRNA (cDNA clone IMAGE:3989958) | C1qtnf3 | 13.6 | 0.041433 |
| CD40 antigen (Cd40), transcript variant 5, mRNA | Cd40 | 12.88 | 0.049520 |
| Arylsulfatase K (Arsk), mRNA | Arsk | 12.54 | 0.009349 |
| Carboxypeptidase A6, mRNA (cDNA clone MGC:141281 IMAGE:40057538) | Cpa6 | 11.99 | 0.005280 |
| Collagen, type VII, alpha 1 (Col7a1), mRNA | Col7a1 | 10.78 | 0.031445 |
| Secreted frizzled-related protein 1, mRNA (cDNA clone MGC:37430 IMAGE:4982195) | Sfrp1 | 10.55 | 0.019490 |
| TNF receptor family member SOBa | Tnfrsf22 | 10.31 | 0.029456 |
| Periostin, osteoblast specific factor, mRNA (cDNA clone MGC:25368 IMAGE:4457222) | Postn | 10.1 | 0.010315 |
| Nidogen 1 (Nid1), mRNA | Nid1 | 9.6 | 0.019519 |
| Tolloid-like (Tll1), mRNA | Tll1 | 9.13 | 0.017271 |
| Interleukin 7 (Il7), mRNA | Il7 | 7.85 | 0.018148 |
| Matrix Gla protein, mRNA (cDNA clone MGC:46939 IMAGE:4990627) | Mgp | 7.7 | 0.009075 |
| Lysyl oxidase-like 1, mRNA (cDNA clone MGC:46960 IMAGE:5251968) | Loxl1 | 7.65 | 0.011397 |
| Angiopoietin 3 (Ang3) | Angpt4 | 6.76 | 0.030365 |
| Ectonucleotide pyrophosphatase/phosphodiesterase 1 allotype b (Enpp1) mRNA, Enpp1-b allele | Enpp1 | 6.54 | 0.048678 |
| **Gene Name** | **Gene ID** | **Ratio** | **adj. *P*-value** |
| Serine (or cysteine) peptidase inhibitor, clade E, member 1 (Serpine1), mRNA | Serpine1 | 6.51 | 0.041746 |
| Ectonucleotide pyrophosphatase/phosphodiesterase 2, mRNA (cDNA clone MGC:6665 IMAGE:3499038) | Enpp2 | 6.39 | 0.015268 |
| Serine (or cysteine) peptidase inhibitor, clade F, member 1, mRNA (cDNA clone MGC:29977 IMAGE:5123884) | Serpinf1 | 6.38 | 0.014596 |
| Capping protein (actin filament), gelsolin-like, mRNA (cDNA clone MGC:28153 IMAGE:3983574) | Capg | 5.61 | 0.015437 |
| Transcribed locus, weakly similar to XP_001473904.1 PREDICTED: hypothetical protein [Mus musculus] | - | 5.56 | 0.032162 |
| Tumor necrosis factor receptor superfamily, member 11b (osteoprotegerin) (Tnfrsf11b), mRNA | Tnfrsf11b | 5.54 | 0.029519 |
| **Insulin-like growth factor binding protein 4, mRNA (cDNA clone MGC:29917 IMAGE:5123738)[9]** | **Igfbp4** | **5.37** | **0.040511** |
| **Fibroblast growth factor 1, mRNA (cDNA clone MGC:46904 IMAGE:5137246)[65]** | **Fgf1** | **4.82** | **0.021812** |
| Microfibrillar associated protein 5, mRNA (cDNA clone MGC:35969 IMAGE:3982519) | Mfap5 | 4.68 | 0.043773 |
| Glypican 4, mRNA (cDNA clone MGC:11506 IMAGE:3967797) | Gpc4 | 4.61 | 0.015437 |
| **Tissue inhibitor of metalloproteinase 1, mRNA (cDNA clone MGC:6151 IMAGE:3158222)[66]** | **Timp1** | **4.57** | **0.039480** |
| Protease, serine, 23 (Prss23), mRNA | Prss23 | 4.49 | 0.030060 |
| Collagen, type XII, alpha 1 (Col12a1), mRNA | Col12a1 | 4.16 | 0.026074 |
| Extracellular matrix protein 1 (Ecm1), mRNA | Ecm1 | 4.14 | 0.020498 |
| Growth hormone receptor, mRNA (cDNA clone IMAGE:4976030) | Ghr | 4.1 | 0.026106 |
| Neuron navigator 2, mRNA (cDNA clone IMAGE:9087590) | Nav2 | 4.09 | 0.009101 |
| Chemokine (C-X3-C motif) ligand 1, mRNA (cDNA clone MGC:5859 IMAGE:3498747) | Cx3cl1 | 3.99 | 0.010546 |
| **Tissue inhibitor of metalloproteinase 2 (Timp2), mRNA[66]** | **Timp2** | **3.93** | **0.032080** |
|  |  |  |  |
| **Gene Name** | **Gene ID** | **Ratio** | **adj. *P*-value** |
| Wingless-related MMTV integration site 5A, mRNA (cDNA clone MGC:27837 IMAGE:3487288) | Wnt5a | 3.79 | 0.034647 |
| **Platelet-derived growth factor, D polypeptide, mRNA (cDNA clone MGC:31518 IMAGE:4489485)[9]** | **Pdgfd** | **3.69** | **0.049537** |
| Energy homeostasis associated, mRNA (cDNA clone IMAGE:5097248) | Enho | 3.31 | 0.033947 |
| Epidermal growth factor-containing fibulin-like extracellular matrix protein 2, mRNA (cDNA clone MGC:18729 IMAGE:3980048) | Efemp2 | 3.28 | 0.037002 |
| Coagulation factor XIII, A1 subunit (F13a1), mRNA | F13a1 | 2.95 | 0.049820 |
| Plasma glutamate carboxypeptidase, mRNA (cDNA clone MGC:46857 IMAGE:4989536) | Pgcp | 2.95 | 0.042904 |
| Matrix metalloproteinase 19 | Mmp19 | 2.85 | 0.048526 |
| ESOP1 (Esop1) | Ly96 | 2.8 | 0.015093 |
| **Gelsolin (Gsn), mRNA[67]** | **Gsn** | **2.63** | **0.038806** |
| Sema domain, immunoglobulin domain (Ig), short basic domain, secreted, (semaphorin) 3E (Sema3e), mRNA | Sema3e | 2.62 | 0.032850 |
| Fibrinogen-like protein 2, mRNA (cDNA clone MGC:19044 IMAGE:4189071) | Fgl2 | 2.61 | 0.016671 |
| Procollagen C-endopeptidase enhancer protein (Pcolce), mRNA | Pcolce | 2.6 | 0.030060 |
| Multiple coagulation factor deficiency 2 (Mcfd2), transcript variant 2, mRNA | Mcfd2 | 2.52 | 0.042345 |
| High density lipoprotein (HDL) binding protein, mRNA (cDNA clone MGC:36446 IMAGE:5354293) | Hdlbp | 2.46 | 0.046796 |
| Collagen, type XVI, alpha 1, mRNA (cDNA clone IMAGE:5342460) | Col16a1 | 2.38 | 0.013452 |
| Apolipoprotein B48 receptor, mRNA (cDNA clone MGC:28748 IMAGE:4482457) | Apob48r | 2.11 | 0.045721 |
| Chitinase domain containing 1, mRNA (cDNA clone MGC:6792 IMAGE:2647540) | Chid1 | 2.08 | 0.044922 |
